# Flow Channel Influence of a Collision-Based Piezoelectric Jetting Dispenser on Jet Performance

**DOI:** 10.3390/s18041270

**Published:** 2018-04-20

**Authors:** Can Zhou, Guiling Deng, Junhui Li, Ji’an Duan

**Affiliations:** 1 School of Mechanical and Electronical Engineering, Central South University, No. 932, South Lushan Road, Changsha 410083, China; zhoucan@csu.edu.cn; 2 School of Information Science and Engineering, No.932, South Lushan Road, Changsha 410083, China; 3 State Key Laboratory of High Performance Complex Manufacturing, No. 932, South Lushan Road, Changsha 410083, China

**Keywords:** flow channel influence, bi-piezoelectric jet, mechanical-electrical-fluid simulation, microelectronics packaging

## Abstract

To improve the jet performance of a bi-piezoelectric jet dispenser, mathematical and simulation models were established according to the operating principle. In order to improve the accuracy and reliability of the simulation calculation, a viscosity model of the fluid was fitted to a fifth-order function with shear rate based on rheological test data, and the needle displacement model was fitted to a nine-order function with time based on real-time displacement test data. The results show that jet performance is related to the diameter of the nozzle outlet and the cone angle of the nozzle, and the impacts of the flow channel structure were confirmed. The approach of numerical simulation is confirmed by the testing results of droplet volume. It will provide a reliable simulation platform for mechanical collision-based jet dispensing and a theoretical basis for micro jet valve design and improvement.

## 1. Introduction

The mechanical collision-based jetting dispenser is a significant device in the electronic information manufacturing industry, as it features high performance and high efficiency [1,2,3,4,5,6].

**Figure 1:**
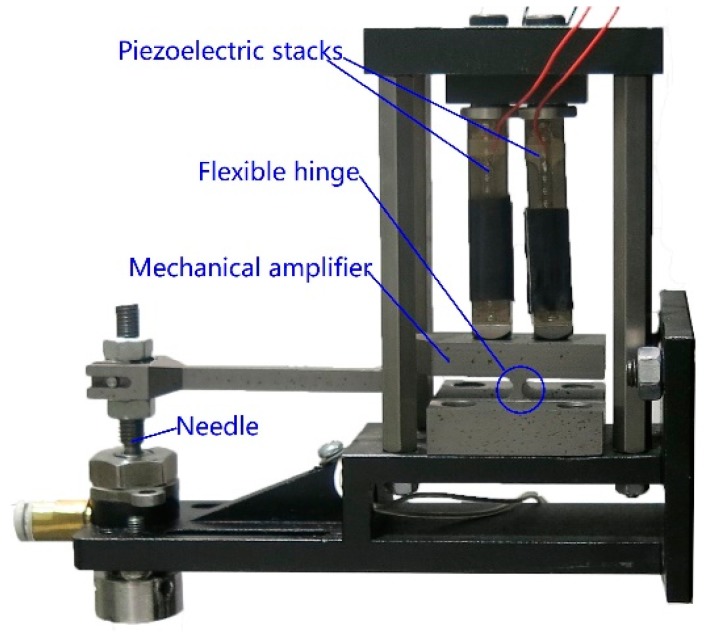
Labelled image of a bi-piezo stacks-driven jet dispenser.

In the 1970s and 1980s, many experts studied ink-jet technology [7,8,9,10], but this technology is only applicable for low viscosity (<10 mPa·s) fluid [11]. The piezoelectric jet dispensing technology for high viscosity fluid was first proposed in 2007 [12]. After that, a great deal of research about jetting valves or droplet-forming theory have been published [13,14,15]. Wang designed a piezoelectric jet dispenser and studied the jet performance [16,17]. Ahamed proposed a non-contact drop-on-demand three-layer micro droplet generator based on electrostatic actuation [18]. Lu proposed a piezoelectric impinging jet valve to investigate the effect of different structural parameters on performance [19]. Many other experts have devoted their energies to jet dispensers and made great achievements [20,21,22,23,24,25]. A bi-piezo stacks-driven jet dispenser was proposed in a previous study [26]. The bi-piezo stacks-driven jet dispenser is shown in Figure 1. The piezoelectric jet dispenser stacks work alternatively. The needle moves back and forth with the lever of the mechanical amplifier. The fluid inflow channel flows with the needle, and pressure forms. The structure and dimension of the flow channel determines the pressure. The pressure makes droplets jet out from the nozzle. The work frequency of the proposed jet dispenser can reach 500 Hz. The dispensers have achieved encouraging results, but the flow channel influence of a novel bi-piezoelectric jet valve on jet performance has not been studied.

Du studied the jetting dispenser, driven by mechanical collision, with simulations and experiments [27]. Wu also studied the droplet-forming process and designed an experimental jet device [28]. However, in their studies, the displacement of the needle was simplified as a trapezoidal wave, and the adhesives were assumed to be a power law fluid. In this study, the displacement of the needle and the fluid viscosity are tested, and the results are used in a finite element analysis to improve accuracy.

A mechanical collision-driving droplet formation model has been proposed. The tested displacement of the needle and the tested characteristics of adhesives have been applied as boundary conditions in the simulation model. A corresponding jet device has been designed to verify the model. Furthermore, the effects of the flow channel and the optimization are discussed. This study will provide a theoretical basis for the design of a mechanical collision-based jet dispenser.

## 2. Bi-Piezoelectric Jet Valve-Based Jet Dispensing Experimental Platform

**Figure 2:**
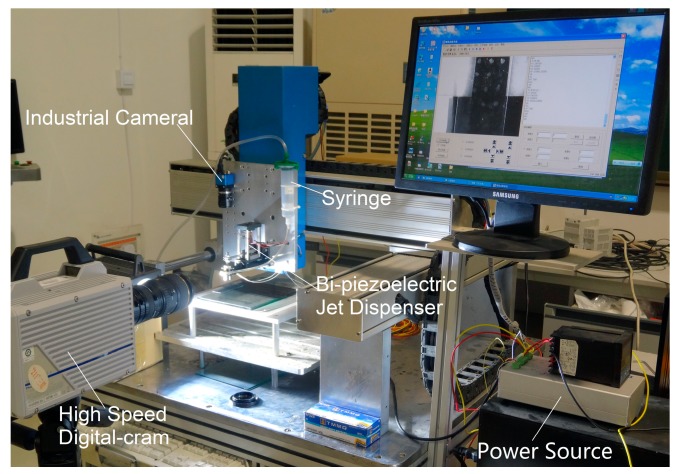
The mechanical collision-based jet dispensing experiment platform.

In this study, a bi-piezoelectric jet dispenser was designed, and the corresponding experimental platform was established. The experimental system consisted of a jet dispenser, a graduated cylinder, a High Speed Digital-Cram (brand: Photron, model: FastCAM Sa1.1 (Photron Limited, Tokyo, Japan)), a rheometer (Malvern Kinuex (Malvern Panalytical, Malvern, UK)), a laser displacement sensor (Kyence LK-G80 (Kyence Corporation, Osaka, Japan)), an industrial camera (Myutron-FV2020 (Myutron Inc., Tokyo, Japan)), an XYZ-axis motion platform, and other accessory devices, as presented in Figure 2 [26].

The piezoelectric jet dispenser is driven by two piezoelectric stacks and a lever principle-based mechanical magnifying device. The displacement of the needle changes with the driving voltage, and the needle displacement of the jet dispenser is measured by the laser displacement sensor, which is fitted to a polynomial, and applied in simulation. Real-time displacement can be measured by a laser sensor. The work process was recorded by a high-speed camera, and the droplet-forming process can be observed through the recorded video. As the droplet volume is not more than 30 nL, it is difficult to test the volume directly. The droplet diameter was measured by an industrial camera, and the droplet volume is represented by the diameter. The average droplet volume was assessed using a graduated cylinder. The fluid characteristics were tested by rheometer, which was used in the simulation.

## 3. Principle and Models of Mechanical Collision-Based Jet Dispensing

**Figure 3:**
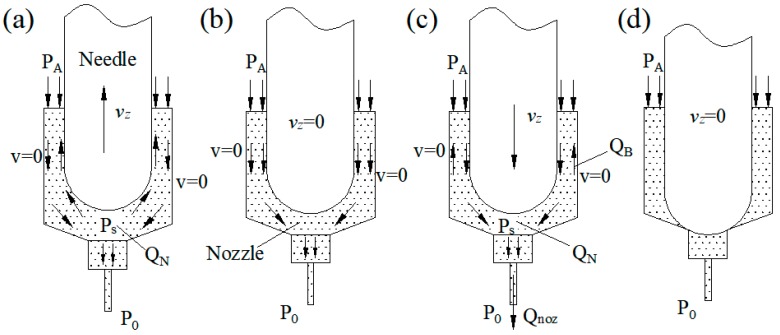
Work flow of mechanical collision-based jet dispensing: (**a**) the needle moves upward; (**b**) the needle stays at the top of the stroke; (**c**) the needle moves downward; (**d**) the needle stays at the bottom of the stroke.

The principle of mechanical collision-based jet dispensing is presented in Figure 3. At the beginning, the needle moves upward, pressure *P*_s_ is smaller than *P*_A_, and fluid flows into the nozzle. The fluid flow direction of the process is presented in Figure 3a. After reaching the top of the stroke, the needle will stop for several microseconds, so that the fluid can fully fill the nozzle chamber. In this process, *v*_z_ = 0 and *P*_A_ = *P*_s_. Then, the needle moves rapidly downward, fluid around the needle flows with the needle, and pressure in the nozzle forms. Fluid in the chamber will be jetted out. In this process, as *P*_A_ < *P*_s_, part of the fluid in the chamber flows backward. The fluid flowing direction of the process is presented in Figure 3c. The needle stays at the bottom of the stroke for a period of time that is dependent on the work frequency. In this process, the needle matches the nozzle to prevent fluid leaking. After this step, the next cycle follows. The needle reciprocates in the nozzle chamber at high speed. Fluid in the chamber can jet out on demand.

### 3.1. Properties of the Fluid

Mechanical collision-based jet dispensing is sensitive to fluid properties, especially viscosity. There are many types of adhesives used in the packaging industry. Most adhesives are non-Newtonian fluids. However, adhesives are usually simplified as Newtonian fluids or power law fluids in jet dispensing studies. To improve calculation accuracy, the properties of adhesives are tested with rheometers. The results are polynomial-fitted and applied in simulation models.

**Figure 4:**
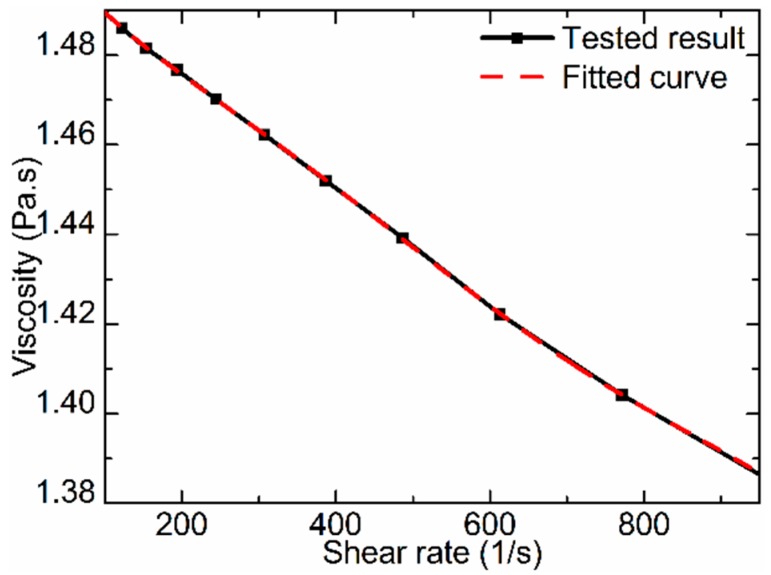
Fluid properties.

The viscosity of a fluid is one of the key factors affecting the jet performance of mechanical collision-based jet dispensing. Adhesives used in the experiment were tested with a Malvern Kinuex rheometer, at 25 °C. The results were fitted to a fifth-order polynomial. The tested property of the selected adhesives and the fitted results are presented in Figure 4. The fitted polynomial is shown as Equation (1).

(1) $$\begin{array}{cc} \eta = & 1.50856-2.35561\times 10^{-4}r^{\prime }+5.73593\times 10^{-7}{r^{\prime }}^{2}-1.35002\times 10^{-9}{r^{\prime }}^{3}+1.41032\times 10^{-12}{r^{\prime }}^{4}\\ & -5.26051\times 10^{-16}{r^{\prime }}^{5} \end{array}$$

where *η* is the viscosity and *r*′ is the shear rate.

### 3.2. Needle Displacement

**Figure 5:**
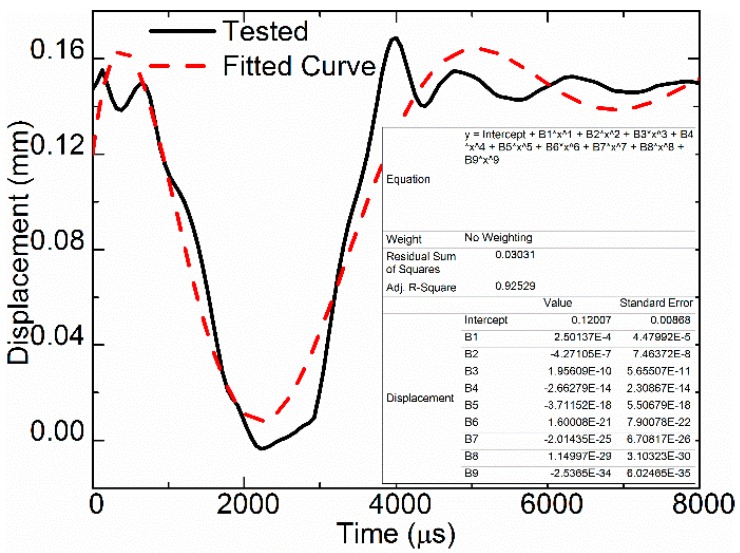
Needle displacement.

Some experts simplify the needle displacement as a sinusoidal wave in simulation models. This reduces the accuracy of the simulation. With the following parameters of voltage = 120 V, duty circle = 0.3, and frequency = 100 Hz, the needle displacement was tested by a laser displacement sensor, and the results were fitted to a ninth-order polynomial. The tested displacement and the fitted curve are presented in Figure 5. The ninth-order polynomial cannot depict the tested results accurately. Therefore, the needle displacement was obtained through an interpolation algorithm in the simulation model.

(2) $$\begin{array}{cc} s= & 0.12007+2.50137\times 10^{-4}t-4.27105\times 10^{-7}t^{2}+1.95609\times 10^{-10}t^{3}-2.66279\times 10^{-14}t^{4}\\ & -3.71152\times 10^{-18}t^{5}+1.60008\times 10^{-21}t^{6}-2.01435\times 10^{-25}t^{7}+1.14997\times 10^{-29}t^{8}\\ & -2.5365\times 10^{-34}t^{9} \end{array}$$

where *s* is the displacement and *t* is time.

### 3.3. Fluid Models

The assumption is that fluid in the nozzle is continuous and impressable when the needle is moving. Fluid in the nozzle chamber follows the continuity and Navier-Stokes law.

The outlet of the nozzle was a 0.1-mm diameter fine pipe. Fluid flowing through the fine pipe follows the Hagen–Poiseuille law. Flow resistance of the nozzle outlet is inversely proportional to the fourth power of radius, i.e., it is proportional to the length of the outlet and the fluid viscosity coefficient. The flow can be expressed as Equation (4):

(3) $$Q=\frac{\pi r^{4}\cdot \Delta P}{8\eta l}$$

where *Q* is flow, *r* is the radius of the nozzle outlet, ∆*P* is the pressure difference between the inlet and outlet, *η* is viscosity coefficient, and *l* is the length of the outlet.

### 3.4. Simulation Model

**Figure 6:**
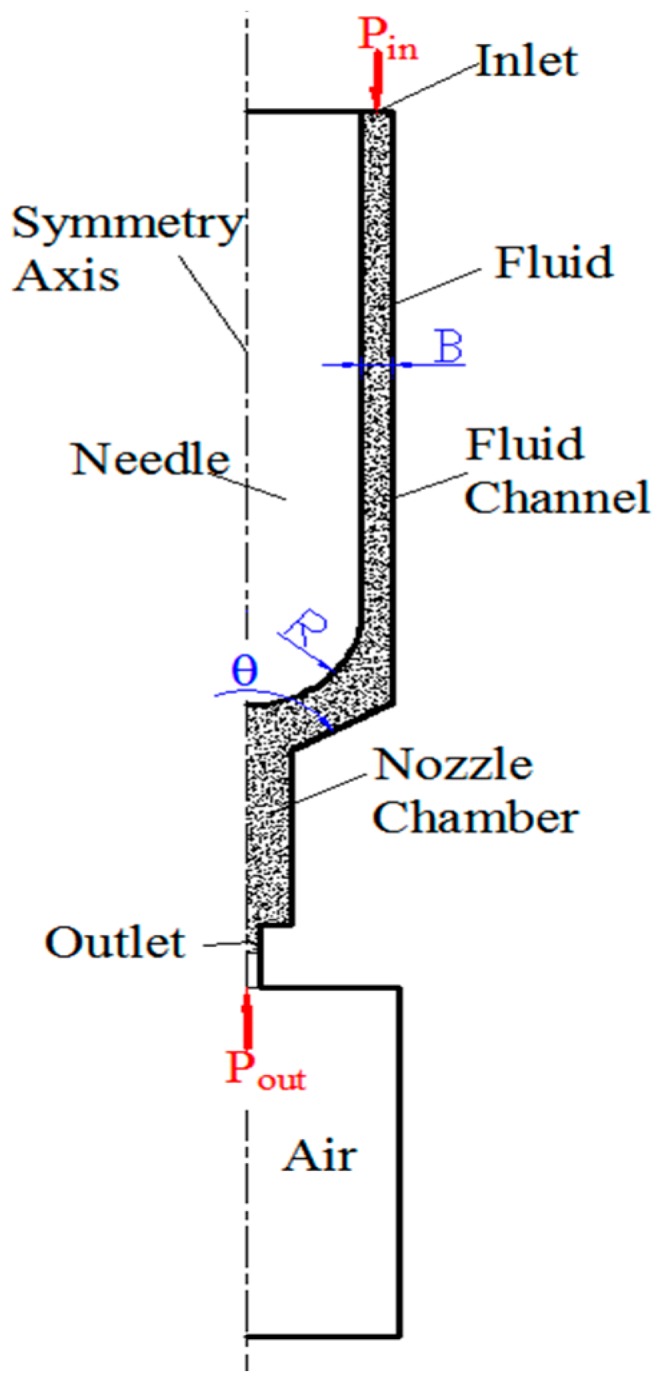
Symmetrical geometry model of the simulation.

The jet dispensing system, based on the mechanical collision system, was simplified as an axis symmetric model. The simulation geometry model is shown in Figure 6. The model was established and meshed with ICEM (Integrated Computer Engineering and Manufacturing code for Computational Fluid Dynamics). Then, the meshes were exported and imported into Fluent.

The moving meshes were analyzed with the self-programed UDF (user defined function) where the needle was a moving part. The displacement of the meshes was defined as the interpolation of the tested results. The fluid property was also defined as the fitted polynomial (Equation (1)) using UDF. The filling pressure (*P_in_*) at the inlet was 0.4 MPa, and the pressure at outlet (*P_out_*) was the atmospheric pressure (0.1 MPa). The needle radius was *R* = 0.75 mm, and the end of the needle was a hemisphere. The fluid around the needle flowed with the needle, and the flow velocity was equal to the needle-moving velocity. Velocity of the fluid near the nozzle wall was zero. In the simulation model, the temperature variations were ignored.

## 4. Results and Discussion

**Figure 7:**
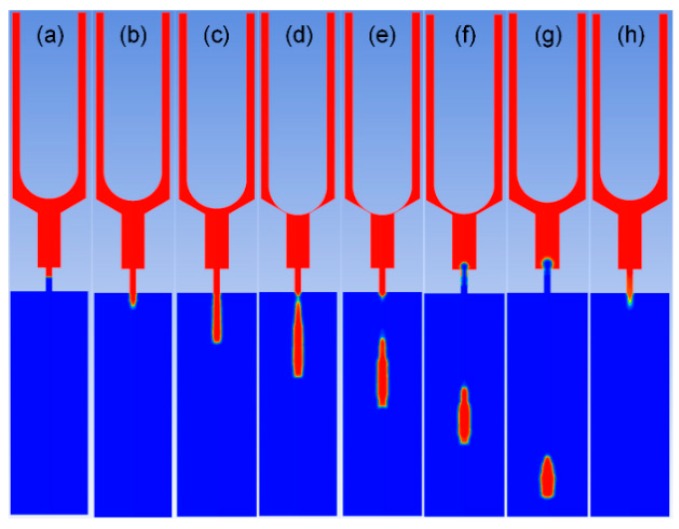
Volume fraction contour: (**a**) the beginning of a jetting process; (**b**) the needle starts moving downward; (**c**) fluid is just jet out from the nozzle; (**d**) the filament breaks off; (**e**,**g**) the droplet moves downward; (**f**) the needle starts moving upward; (**h**) the end of the cycle.

**Figure 8:**
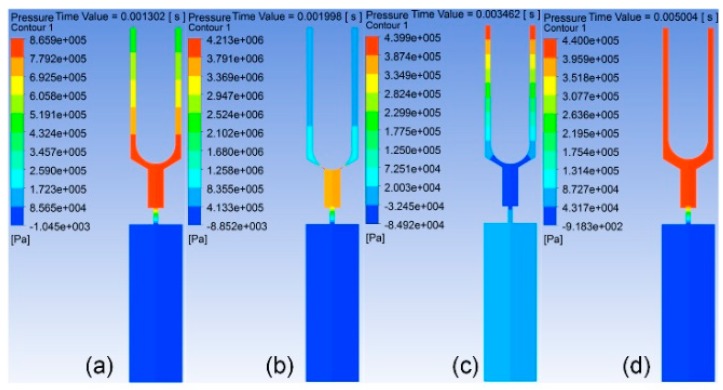
Pressure contour: (**a**) the pressure contour when the needle is moving down; (**b**) the pressure contour while the needle strikes the nozzle; (**c**) the pressure contour while the needle moves up; (**d**) the pressure contour while the needle is at the top of the stroke.

To study the impact of flow channel on jet performance, the simulation and experiment were carried out. The volume fraction contour figures during the work processes are presented in Figure 7, and the pressure contour figures during the work processes are presented in Figure 8. According to the operating principle of the jetting dispenser, the working process consists of three stages.

In the first stage, the needle moves from the highest position to the lowest position and collides with the inner wall of the nozzle. Figure 7a–e presents the simulation results of the volume fraction contour of this stage, and Figure 8a,b presents the simulation results of the pressure contour of this stage. In this process, fluid flows with the needle, and pressure at the nozzle chamber is gradually increased. When the needle strikes on the inner wall of the nozzle, pressure at the nozzle chamber reaches a maximum. Part of fluid in the pressure chamber is jet out from the nozzle under the pressure. At the same time, the pressure in the pressure chamber decreases.

In the second stage, the needle starts to move to the upper position from the lowest position. Figure 7f,g presents the volume fraction contour of this stage, and Figure 8c presents the pressure contour of this stage. With the needle moving upward, the volume of the pressure chamber increases. Fluid then flows into the chamber with filling pressure. As the volume of fluid filling into the chamber is less than the space formed by the needle moving, negative pressure forms in the chamber, so that fluid in the nozzle outlet begins to flow back. This will accelerate the filament breaking off and droplet formation.

In the third stage, the needle stops at the highest position, where the filling pressure is greater than the pressure in the nozzle chamber. Fluid will fill into the chamber from the inlet until the chamber is full of fluid or the next cycle begins. Figure 7h presents the volume fraction contour of this stage, and Figure 8d presents the pressure contour of this stage.

**Figure 9:**
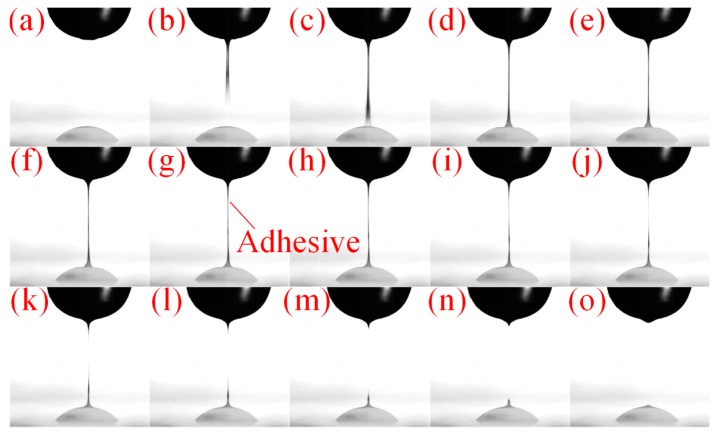
Droplet formation process: (**a**) the beginning of the cycle; (**b**) the fluid is jet out from the nozzle; (**c**) the fluid jet from the nozzle strikes the substrate; (**d**–**h**) the neck of the filament appears; (**i**–**k**) the radius of the filament becomes extremely small and the filament breaks off; (**l**–**n**) the bottom droplet forms a droplet and the top filament recoils back to the nozzle due to negative pressure in the nozzle chamber and surface tension; (**o**) the end of the cycle.

The work process is recorded by a High Speed Digital-Cram when the experiment is carried out, which is presented in Figure 9 [26].

### 4.1. Impacts of Nozzle Outlet Diameter

**Figure 10:**
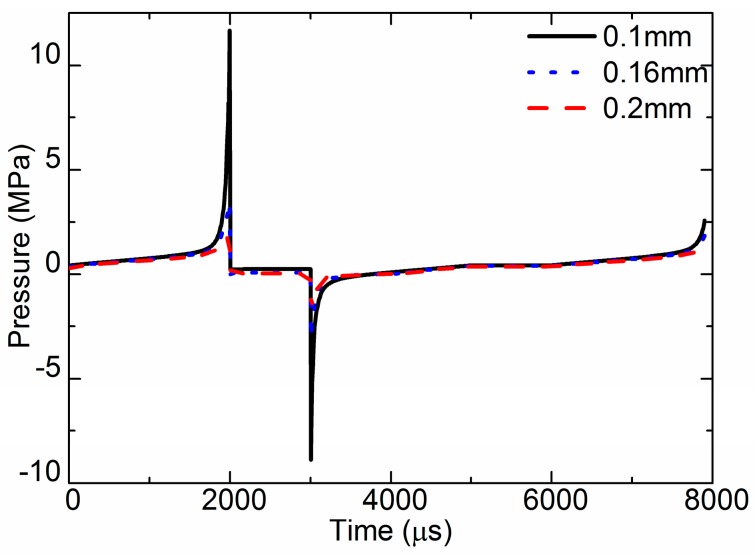
Pressure at nozzle outlet of the different nozzle diameters.

For a different requirement, the diameter of the nozzle outlet may be different. The simulated results of pressure curves are presented in Figure 10. It is obvious that the pressure-varying trends of the three different size nozzles were similar during the jet process, but the amplitude of the pressure was different. When the diameter was 0.1 mm, the max pressure was 11.8 MPa, and the max negative pressure was −8.98 MPa. When the diameter was 0.16 mm, the max pressure was 3.58 MPa. When the diameter was 0.2 mm, the max pressure was 1.92 MPa. The smaller the diameter of the nozzle outlet, the higher the pressure was, because fluid leaking from the small outlet was difficult when the needle moved.

**Figure 11:**
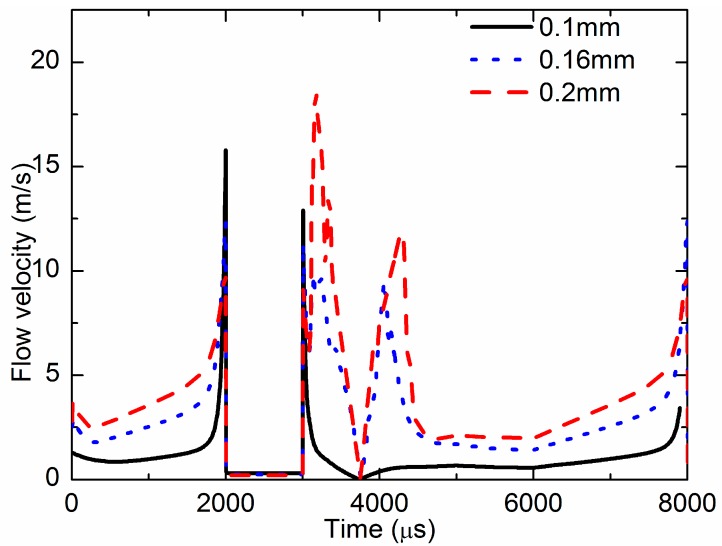
Flow velocity at the nozzle outlet of the different nozzle diameters.

The flow velocity was the key factor that determined if fluid could be jetted out and form a droplet. Figure 11 demonstrates the simulated flow velocity curves at the nozzle outlet for different size nozzles. The curves present the value of the flow velocity, but not the direction. There are two peaks in the curve during one cycle. The first peak is the flow velocity when the needle strikes the nozzle, which affects the jet performance. The second peak is the flow velocity when the needle begins moving upward, and the flow direction is upward. When the diameter of the nozzle outlet was 0.1 mm, the max flow velocity was 15 m/s, and the max velocity of flow back was 13 m/s. When the diameter of the nozzle outlet was 0.16 mm, the first peak value was 12.5 m/s, and the max velocity of flow back was 10 m/s. When the diameter of the nozzle outlet was 0.16 mm, the first peak value was 10 m/s, and the second peak value was 18.5 m/s. The smaller the outlet, the larger the flow velocity at the outlet when the needle strikes the nozzle.

**Figure 12:**
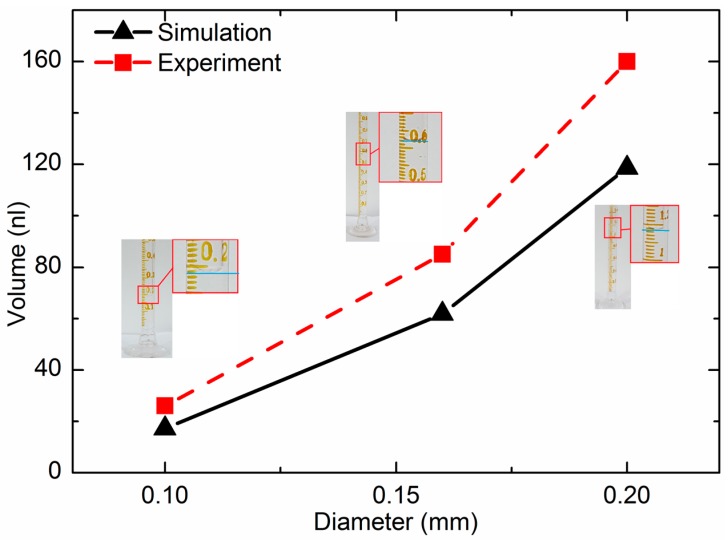
Droplet volume of the different radius comparing experimental and simulation results.

Droplet volume is a key parameter of jet performance, which must meet the requirements of different industries. The simulation results were evaluated by an image-processing method. The droplet image is shown in Figure 7. Assuming that the droplet was axisymmetric, the droplet can be observed as a number of cylinder stacks. The volume of the droplet can be calculated as the volume summary of a number of one pixel high cylinders, approximately. In the experiment, the volume of ten thousand droplets was tested with a graduated cylinder. Then, the average volume of the droplet could be calculated. Figure 12 presents the relationship between the droplet volume and the size of nozzle outlet. When the cone angle was 120°, the channel width was 0.1 mm, and when the diameters of the nozzle outlet were 0.1 mm, 0.16 mm, and 0.2 mm, the volume of the droplet was approximately 19 nL, 62 nL, and 119 nL, respectively. The droplet volume increased with the diameter of the nozzle outlet.

### 4.2. Impacts of Cone Angle

**Figure 13:**
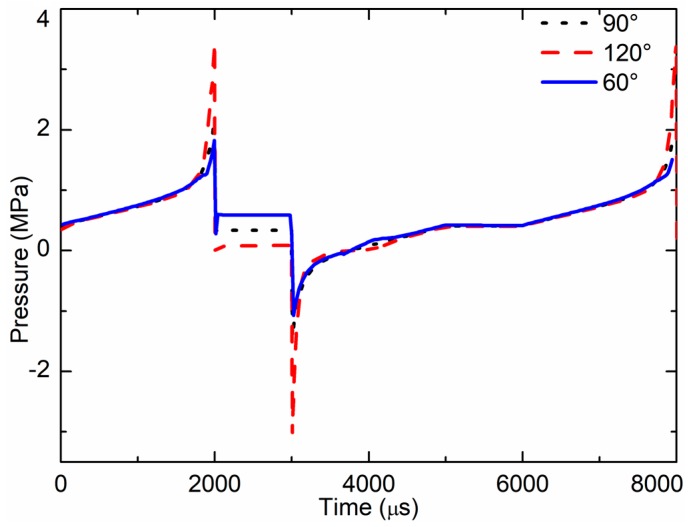
Pressure at nozzle outlet for the different cone angles.

The cone angle of the nozzle is marked as *θ* in Figure 6. As the cone angle might affect the jet performance of the micro jet valve, it is an important parameter in jet valve design. The impacts of cone angles on jet pressure, flow velocity, and droplet volume were studied. The simulated results of pressure at the nozzle outlet of one cycle are shown in Figure 13. When the cone angle was 120°, the max pressure at the nozzle outlet was 3.2 MPa, and the max negative pressure was −3.2 MPa. When the cone angle was 90°, the max pressure at the nozzle outlet was 2 MPa, and the max negative pressure was −1.8 MPa. When the cone angle was 60°, the max pressure at the nozzle outlet was 1.82 MPa, and the max negative pressure was −1.07 MPa. The pressure of the 120° cone angle nozzle was greater than that of the 60° and 90° cone angles.

**Figure 14:**
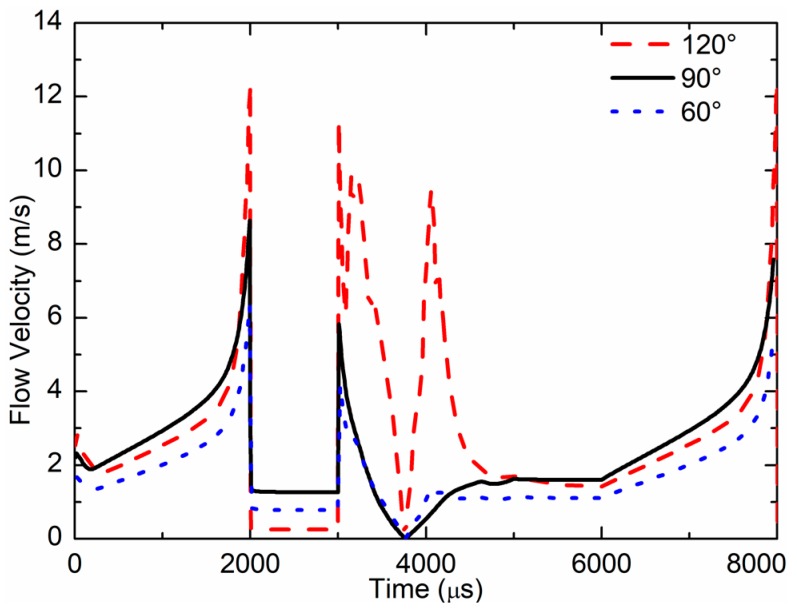
Flow velocity at nozzle outlet for the different cone angles.

The simulated results of flow velocity at the nozzle outlet during one cycle are shown in Figure 14. The max flow velocity of the 120° cone angle nozzle was 12.5 m/s, the max flow velocity of the 90° cone angle nozzle was 8.5 m/s, and the max flow velocity of the 60° cone angle nozzle was 6.36 m/s. The flow velocity of the 120° cone angle nozzle was larger than that of the 60° and 90° cone angles.

The impact of the cone angle on droplet volume was also assessed. When the nozzle diameter was 0.16 mm, the channel width was 0.1 mm, and when the cone angles were 60°, 90° and 120°, the volume of the droplet was approximately 122 nL, 106 nL, and 62 nL, respectively. The results are presented in Table 1. The volume of the droplet jet by the nozzle of the 120° cone angle was smaller than that jet by the nozzle of the 60° and 90° cone angles.

### 4.3. Impacts of the Channel Width

**Figure 15:**
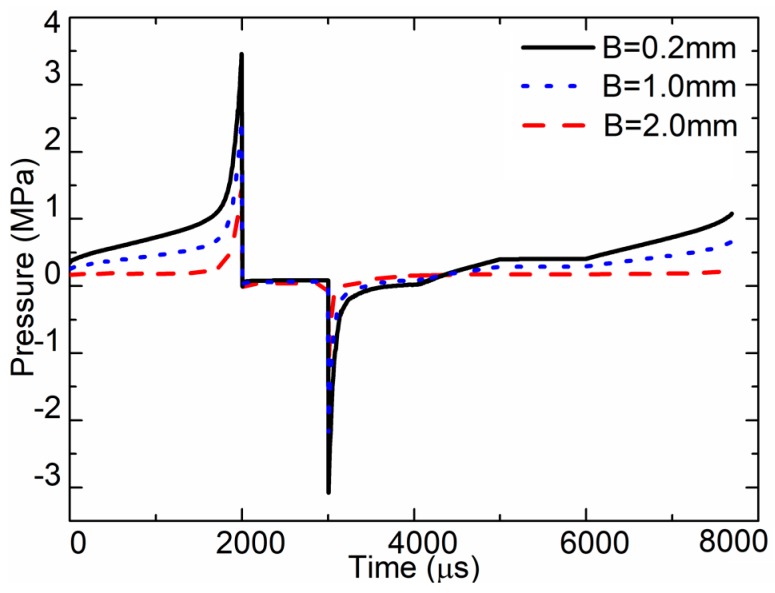
Pressure at nozzle outlet for the different channel widths.

The flow channel is the gap between the needle and the nozzle. The width of the channel is marked as B in Figure 6. The impacts of the channel width were also studied, and the simulated pressure curves at the nozzle outlet of different channel widths are presented in Figure 15. When B = 2.0 mm, the max pressure in the jet stage was approximately 1.5 MPa. When B = 1.0 mm, the max pressure in the jet stage was approximately 2.48 MPa. When B = 0.2 mm, the max pressure in the jet stage was approximately 3.5 MPa. When the gap is larger, there will be more fluid flowing back in the channel during the jet stage.

**Figure 16:**
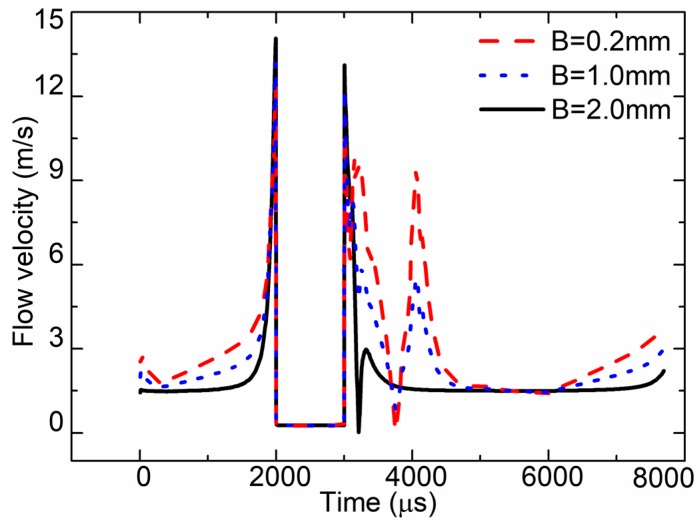
Flow velocity at nozzle outlet for the different channel widths.

Figure 16 demonstrates the simulated flow velocity curve at the nozzle outlet of different channel widths. When B = 2.0 mm, the max flow velocity in the nozzle outlet was approximately 12.3 m/s. When B = 0.2 mm, the max flow velocity in the nozzle outlet was approximately 14 m/s. When B = 1.0 mm, the max flow velocity in the nozzle outlet was approximately 13.2 m/s.

The impact of the channel width on the droplet volume was also assessed. When the nozzle diameter was 0.16 mm, the cone angle was 120°, and when the channel width was 0.2 mm, 1.0 mm, and 2.0 mm, the volume of the droplet was approximately 39 nL, 62 nL, and 75 nL, respectively. The results are presented in Table 2. The droplet volume of the channel width of 2.0 mm was larger than that of 1.0 mm and 0.2 mm. The larger channel width, the more fluid filled into the channel during the same time.

## 5. Conclusions

The influence laws of flow channel structure were studied through simulation and experimentation. (1) For the jetting dispensing process, when the needle moves from the above to the lowest position, the chamber pressure reaches its maximum value, and the smaller the diameter of the nozzle is, the greater the maximum pressure value will be; (2) the flow velocity and pressure of the 120° cone angle nozzle was larger than the 90° cone angle nozzle, and the droplet volume of the 120° cone angle nozzle was smaller than the 90° cone angle nozzle; (3) while the channel width increases, the flow velocity and pressure decrease, and the droplet volume increases.

Furthermore, the tested fluid properties and needle displacement-based simulation model will provide a theoretical analysis platform to develop the micro jet valve.

## Figures and Tables

**Table 1:** Droplet volume for the different cone angles.

Cone Angle (degree)	Droplet Volume	
	Tested (nL)	Simulated (nL)
60	122	115
90	106	94
120	62	57

**Table 2:** Droplet volume for the different channel widths.

Channel Width (mm)	Droplet Volume	
	Tested (nL)	Simulated (nL)
0.2	39	31
1.0	62	57
2.0	75	68
